# Reactivating Immunity Primed by Acellular Pertussis Vaccines in the Absence of Circulating Antibodies: Enhanced Bacterial Control by TLR9 Rather Than TLR4 Agonist-Including Formulation

**DOI:** 10.3389/fimmu.2019.01520

**Published:** 2019-07-03

**Authors:** Floriane Auderset, Marie Ballester, Beatris Mastelic-Gavillet, Paola Fontannaz, Martine Chabaud-Riou, Nathalie Reveneau, Marie Garinot, Noëlle Mistretta, Yuanqing Liu, Paul-Henri Lambert, Martina Ochs, Claire-Anne Siegrist

**Affiliations:** ^1^World Health Organization Collaborating Center for Vaccine Immunology, Departments of Pathology-Immunology and Pediatrics, University of Geneva, Geneva, Switzerland; ^2^Sanofi-Pasteur R&D, Marcy l'Etoile, France

**Keywords:** vaccine, pertussis, adjuvant, TLR9 agonist, TLR4 agonist

## Abstract

Pertussis is still observed in many countries despite of high vaccine coverage. Acellular pertussis (aP) vaccination is widely implemented in many countries as primary series in infants and as boosters in school-entry/adolescents/adults (including pregnant women in some). One novel strategy to improve the reactivation of aP-vaccine primed immunity could be to include genetically- detoxified pertussis toxin and novel adjuvants in aP vaccine boosters. Their preclinical evaluation is not straightforward, as it requires mimicking the human situation where T and B memory cells may persist longer than vaccine-induced circulating antibodies. Toward this objective, we developed a novel murine model including two consecutive adoptive transfers of the memory cells induced by priming and boosting, respectively. Using this model, we assessed the capacity of three novel aP vaccine candidates including genetically-detoxified pertussis toxin, pertactin, filamentous hemagglutinin, and fimbriae adsorbed to aluminum hydroxide, supplemented—or not—with Toll-Like-Receptor 4 or 9 agonists (TLR4A, TLR9A), to reactivate aP vaccine-induced immune memory and protection, reflected by bacterial clearance. In the conventional murine immunization model, TLR4A- and TLR9A-containing aP formulations induced similar aP-specific IgG antibody responses and protection against bacterial lung colonization as current aP vaccines, despite IL-5 down-modulation by both TLR4A and TLR9A and IL-17 up-modulation by TLR4A. In the absence of serum antibodies at time of boosting or exposure, TLR4A- and TLR9A-containing formulations both enhanced vaccine antibody recall compared to current aP formulations. Unexpectedly, however, protection was only increased by the TLR9A-containing vaccine, through both earlier bacterial control and accelerated clearance. This suggests that TLR9A-containing aP vaccines may better reactivate aP vaccine-primed pertussis memory and enhance protection than current or TLR4A-adjuvanted aP vaccines.

## Introduction

*B. pertussis* (Bp), the causative agent of whooping cough, is a gram-negative bacterium highly transmissible in humans across all ages and an important cause of morbidity and mortality in infants worldwide. Introduced in 1950s, whole-cell pertussis (wP) vaccines dramatically reduced disease incidence in infants and young children. However, vaccine-associated reactogenicity and unjustified fears of vaccine-induced encephalopathy affected public confidence and compliance. This lead in the late 1990s to their replacement in most developed countries by less reactogenic acellular pertussis (aP) vaccines (1). Pediatric aP vaccines are composed of 1–5 Bp antigens adsorbed to Alum, combined with diphtheria (DT) and tetanus (TT) toxoids (DTaP) ± polio, *Haemophilus influenzae b* and hepatitis B antigens. Adolescent/adult booster vaccines (Tdap) include lower amounts of DT and Bp antigens.

Over the last decade, a significant increase of pertussis incidence was observed primarily in aP vaccine-using countries, even despite high coverage in infants and young children (2, 3). This resurgence affects all age groups, but mostly adolescents (2). Several factors may account for the limited durability of aP vaccine effectiveness (4). Among them, aP vaccine immunogenicity is short lasted and protection wanes rapidly over time (5–9). Repeated boosters are thus required to maintain and/or reactivate pertussis immunity (10). In mice, aP vaccines induce preferential CD4^+^ Th2 cell responses (associated to mostly IgG1 antibodies) which significantly differ from the better protective Th1/Th17 responses [associated with IgG2a/b/c antibodies (11)] induced by natural infection or wP immunization (12–14). This Th2 vs. Th1/Th17 pattern appears similar in humans (15–20), in whom both aP and wP vaccines induce IgG1 antibodies while the Th2-associated IgG4 isotype was only observed in aP-vaccinated children (21, 22).

Should the control of Bp depend upon the presence of Th1/Th17 effector and memory cells, novel pertussis vaccines should thus prime for Th1/Th17 immunity in infancy and/or induce Th1/Th17 booster responses despite the Th2-immunity elicited by aP vaccine priming. One major difference between aP and wP vaccines is their lack of virulence factors and pathogen-associated molecular patterns which generate Th1/Th17-driving pro-inflammatory innate cytokines (23–25). Several murine studies have convincingly shown that adding to or replacing Alum with TLR2 (25), TLR4 (14, 26), TLR7 (27), or TLR9 (13, 28, 29) agonists may generate more protective Th1 responses. Additionally, native pertussis toxin (PT) activates the TLR4 receptor (30, 31), inducing dendritic cell maturation and Th1-driving cytokines (32, 33). However, its chemical detoxification, used in most aP vaccines, both suppresses these immunostimulatory properties (32, 33) and alters 80% of PT epitopes (34), reducing the induction of neutralizing antibodies (35) and likely directing B cell responses toward vaccine-specific rather than pathogen-specific epitopes (34). Thus, priming in infancy with novel Th1/Th17-inducing vaccines including genetically-detoxified (gd)PT should provide better protective efficacy than current aP vaccines (36). However, to demonstrate infant vaccine efficacy and safety and to redevelop multivalent infant vaccines appears to most as a major endeavor.

Alternatively, novel vaccine formulations may prove better at boosting aP vaccines-primed memory than current Tdap vaccines. To circumvent the limitations of preclinical models in which antibodies persist to much higher levels than in humans, we previously reported the usefulness of an adoptive transfer model in which aP-induced memory cells were transferred to naïve recipients prior to boosting with Tdap (37). To address the specific influence of various booster formulations, we subsequently developed a novel model including two consecutive adoptive transfers, memory cells induced by boosting aP-primed cell recipient mice being transferred to naïve recipient mice prior to bacterial challenge. Using this model, we tested three modified (m)Tdap formulations composed of gdPT, filamentous hemagglutinin (FHA), pertactin (PRN), and fimbriae type 2 and 3 (FIM2,3) antigens, adjuvanted with Alum and supplemented or not with TLR4A or TLR9A (Table 1). We show here that this model readily discriminates among TLR agonists-adjuvanted modified Tdap vaccines and identifies TLR9A as more effective than TLR4A against Bp challenge.

**Table 1:** Vaccines used and their antigen components.

**Abbreviation**	**Vaccine**	**Pertussis antigen content (μg)**				**TLR agonist (μg)**	
		PT^*^	**PRN**	**FHA**	**FIM2,3**	**TLR4A**	**TLR9A**
DTwP	D.T.COQ/D.T.P	n.d.	n.d.	n.d.	n.d.		
DTaP	DAPTACEL	10	3	5	5		
Tdap	ADACEL	2.5	3	5	5		
mTdap	N/A	10	5	5	7.5		
mTdap/TLR4	N/A	10	5	5	7.5	5	
mTdap/TLR9	N/A	10	5	5	7.5		250

* *Chemically-detoxified PT in DTwP and DTaP; genetically-detoxified PT in mTdap, mTdap/TLR4, and mTdap/TLR9*.

*DTaP, diphtheria, tetanus, and acellular pertussis*.

*Tdap, reduced DTap*.

*mTdap, modified Tdap*.

## Materials and Methods

### Mice

Adult female CD1 and BALB/cByJ mice were purchased from Charles River (L'Arbresle, France) and kept under specific pathogen free conditions. Mice were used at 6–8 weeks of age. All animal experiments were carried out in accordance with Swiss and European guidelines and approved by the Geneva Veterinary Office and by French Ministry of Higher Education, of Research and Innovation and ethic committee.

### Antigens, Adjuvants, and Immunizations

Mice were primed intra-muscularly (i.m.) with 1/5th of a human dose (50 μl in both hind legs) of DTaP (DAPTACEL, Sanofi-Pasteur Ltd.) containing 10 μg chemically-detoxified PT, 5 μg FHA, 3 μg PRN and 5 μg FIM2 and FIM3 (FIM2,3), TT and DT, or of DTwP (D.T.COQ/D.T.P, Sanofi-Pasteur Ltd.) containing ≥4 I.U. of heat-inactivated Bp, in addition to TT and DT. Recipient mice were boosted i.m. with 1/5th of a human dose (50 μl in both hind legs) of DTwP, Tdap (ADACEL, Sanofi-Pasteur Ltd.) containing 2.5 μg chemically-detoxified PT, 5 μg FHA, 3 μg PRN, and 5 μg FIM2,3, TT and DT, or modified Tdap (mTdap) containing 10 μg gdPT, 5 μg PRN, 7.5 μg FIM2/3, 5 μg FHA, 10 Lf/ml TT, 4 Lf/ml DT and 0,66 mg/ml aluminum hydroxide (AlOH) with 250 μg of TLR9A or 5 μg of TLR4A (mTdap/TLR9A or mTdap/TLR4A, Table 1).

### Fluorospots

Splenic IFNγ, IL-5, or IL-17 cytokine-secreting cells were detected by FluoroSpot assay. Briefly, the membrane of 96-well IPFL-bottomed microplates (Millipore) were coated with rat anti-mouse IFNγ, IL-5, or IL-17 antibodies (PharMingen) at 10 μg/mL, incubated overnight at +4°C, washed and blocked with RPMI containing penicillin-streptomycin, L-Glutamine and β-mercaptoethanol (all from Gibco) and 10% FCS (Hyclone). 1 × 10^6^ freshly isolated splenocytes/well were incubated with a mix of PT (2.5 μg/mL), PRN (5 μg/mL), FIM2,3 (5 μg/mL), and FHA (5 μg/mL) antigens in presence of 10 U/mL of murine IL-2 (Bohringer Mannhein). After 24 (IFNγ and IL-17) or 48 (IL-5) h, biotinylated anti-mouse IFNγ (2 μg/mL), IL-5 (1 μg/mL), or IL-17 (1 μg/mL) antibodies (PharMingen) were added for 2 h at RT. Streptavidin-PE (Southern Biotech) at 1 μg/mL was added for 1 h at RT. The plates were stored at +5°C ± 3°C in the dark until reading. Each spot, corresponding to one IFNγ-, IL-5-, or IL-17-secreting cell, was enumerated with an automatic ELISPOT fluorescent plate reader (Microvision). Results were expressed as number of IFNγ-, IL-5-, or IL-17-secreting cells per 1 × 10^6^ splenocytes.

### Adoptive Transfer

Spleens were harvested 42 days after priming or boosting BALB/cByJ mice. Single cell suspensions were obtained by mechanical disruption and processed for red blood cell lysis. 50 × 10^6^ splenocytes (experimentally defined as optimizing the recall of immune memory, *unpublished data*) in 100 μl were transferred intravenously (i.v.) by retro-orbital injection into naïve BALB/cByJ mice.

### *B. pertussis* Challenge

**Figure 1:**
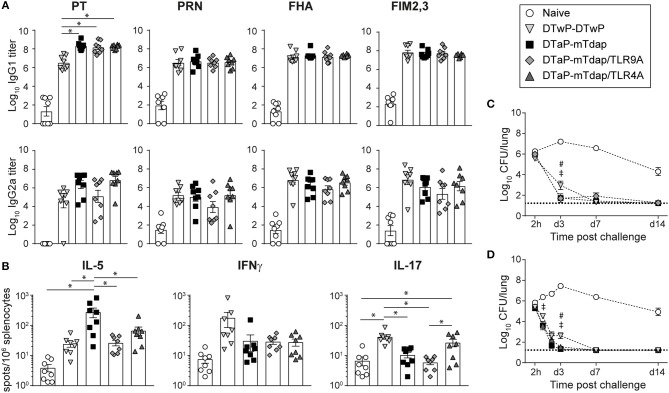
Tdap adjuvantation with TLR agonists exerts little influence on immune and protective responses to pertussis. Adult CD1 mice were immunized i.m. with DTwP or DTaP. 42 days later, DTwP-primed mice were boosted with DTwP while DTaP-primed mice were boosted with mTdap, mTdap/TLR9A, or mTdap/TLR4A. **(A)** PT-, PRN-, FHA-, and FIM2,3-specific IgG1 and IgG2a serum antibody titers were assessed 42 days post boost. The graphs show the mean Log_10_ IgG1 and IgG2a titers (± SEM) of individual mouse sera (*n* = 8). **(B)** Spleens were harvested 42 days post boost. Splenocytes were restimulated with a mix of chemically-detoxified PT, PRN, and FIM2,3 antigens and assessed by fluorospots for IL-5, IFNγ, and IL-17 secretion. The mean numbers of spots (± SEM) per 10^6^ splenocytes for *n* = 8 individual mice are shown. **(C,D)** Mice boosted with DTwP, mTdap, and mTdap/TLR9A **(C)** or mTdap/TLR4A **(D)** were challenged with Bp 42 days post boost. Lungs were harvested 2 h and at days 1, 2 (**D** only), 3, 7, 14, and 21 after challenge to assess bacterial colonization. The graphs indicate the Log_10_ number (± SEM) of CFUs per lung at the indicated time-points for *n* = 12 mice per group. ^*^,#,‡ *P* < 0.05. #DTwP-DTwP vs. DTaP-mTdap; ‡DTwP-DTwP vs. mTdap/TLR9A or TLR4A.

**Figure 2:**
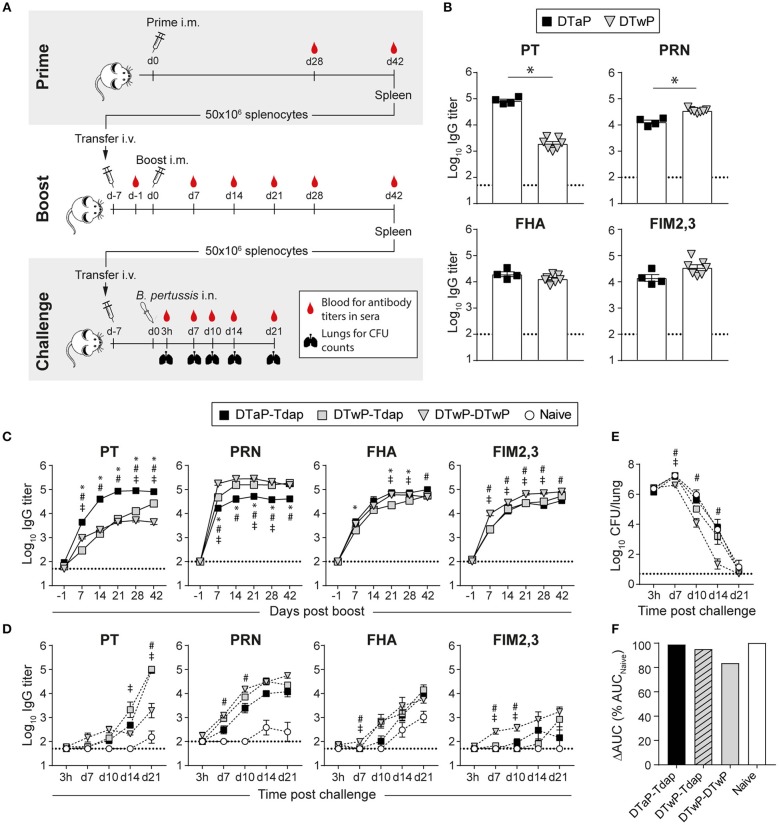
Distinct immunogenicity and protective efficacy of Tdap vs. DTwP in an adoptive transfer model designed to avoid the confounding factor of circulating antibodies. **(A)** BALB/c mice are primed i.m. with DTaP or DTwP and their spleens harvested 6 weeks later for the adoptive transfer of 50 × 10^6^ splenocytes into naïve BALB/c mice. After 6 days, recipient mice are bled to check for the absence of serum pertussis-specific antibodies prior to i.m. boosting. The reactivation kinetics of antigen-specific B cells is evaluated by antibody titers in sera collected at weekly intervals after boosting. Six weeks later, the spleens of boosted recipient mice are harvested and 50 × 10^6^ splenocytes adoptively transferred into naïve BALB/c, 7 days prior to intranasal Bp challenge. Lungs and sera are collected 3 h, and 7, 10, 14, and 21 days after challenge for CFU counting and Ag-specific antibody titers determination, respectively. **(B–D)** BALB/c mice adoptively transferred with 50 × 10^6^ splenocytes of DTaP- or DTwP-primed mice received either Tdap or DTwP boosters. Six weeks later, their splenocytes were adoptively transferred into naïve BALB/c mice prior to intranasal challenge with Bp. PT-, PRN-, FHA-, and FIM2,3-specific IgG antibody responses were assessed in sera collected **(B)** 42 days post prime and at indicated time-points after **(C)** the boost and **(D)** the challenge. The graphs show the mean Log_10_ IgG titers (± SEM) of **(B,C)** pooled (*n* = 4, 6–7 mice per pool) or **(D)** individual sera (*n* = 3–4). The dotted lines indicate the 50% cut-off of the assay. **(E,F)** Lungs were harvested at the indicated time-points after challenge for determination of bacterial colonization. The graphs indicate **(E)** the Log_10_ number of CFUs per lung (± SEM) at indicated time-points for *n* = 3–4 mice per group and **(F)** the area under the clearance curve (AUC) normalized to the AUC from naïve mice. Data are representative of at least two independent experiments. *P* < 0.05 for ^*^DTaP-Tdap vs. DTwP-Tdap; #DTaP-Tdap vs. DTwP-DTwP; ‡DTwP-Tdap vs. DTwP-DTwP.

**Figure 3:**
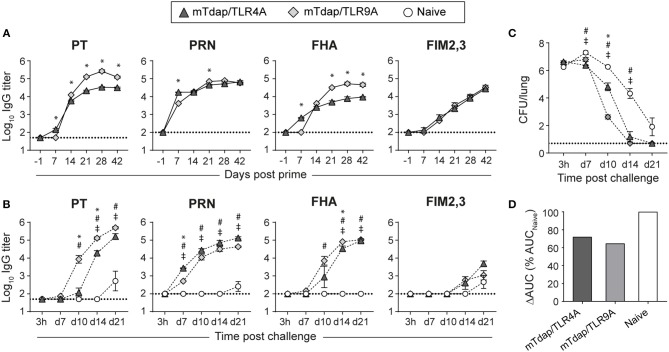
Distinct protective efficacy of a single dose of mTdap/TLR4A and mTdap/TLR9A following bacterial challenge in the absence of serum antibodies. BALB/c mice were immunized i.m. with mTdap/TLR4A or mTdap/TLR9A formulations. Six weeks later, their splenocytes were harvested and adoptively transferred into naïve BALB/c mice challenged with Bp. The kinetics of PT-, PRN-, FHA-, and FIM2,3-specific IgG antibody responses were assessed in sera collected at indicated time-points after **(A)** the prime and **(B)** the challenge. The graphs show the mean Log_10_ IgG titers (± SEM) of **(A)** pooled (*n* = 4, 6–7 mice per pool) or **(B)** individual mouse sera (*n* = 3–4). The dotted lines indicate the 50% cut-off of the assay. **(C,D)** Lungs were harvested 3 h and at days 7, 10, 14, and 21 after challenge and processed for determination of bacterial colonization. The graphs indicate **(C)** the Log_10_ number of CFUs per lung (± SEM) at indicated time-points for *n* = 3–4 mice per group and **(D)** the area under the clearance curve (AUC) normalized to the AUC from naïve mice. Data are representative of at least two independent experiments. *P* < 0.05 for ^*^mTdap/TLR4A vs. mTdap/TLR9A; #mTdap/TLR9A vs. naive; ‡mTdap/TLR4A vs. naive.

**Figure 4:**
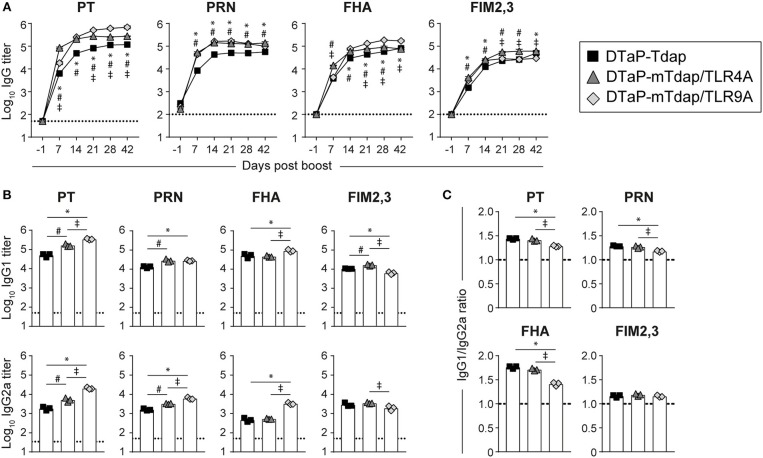
Boosting DTaP-primed mice with mTdap vaccines rapidly and strongly recalls antibody responses to pertussis antigens. BALB/c mice adoptively transferred with 50 × 10^6^ splenocytes of DTaP-primed mice were boosted with Tdap or adjuvanted mTdap formulations. **(A)** The kinetics of PT-, PRN-, FHA-, and FIM2,3-specific IgG antibody responses were assessed in sera collected at the indicated time-points after boosting. The graphs show the mean Log_10_ IgG titers (± SEM) of pooled sera (*n* = 4, 6–7 mice per pool). **(B,C)** IgG1 and IgG2a titers were assessed in sera collected 42 days post boost. The graphs show the **(B)** mean Log_10_ IgG1 and IgG2a titers (± SEM) and **(C)** the IgG1:IgG2a ratio of pooled sera (*n* = 4, 6–7 mice per pool). The dotted lines indicate the 50% cut-off of the assay. Data are representative of at least two independent experiments. *P* < 0.05 for: ^*^DTaP-Tdap vs. DTaP-mTdap/TLR9A; #DTaP-Tdap vs. DTaP-mTdap/TLR4A; ‡DTaP-mTdap/TLR9A vs. DTaP-mTdap/TLR4A.

**Figure 5:**
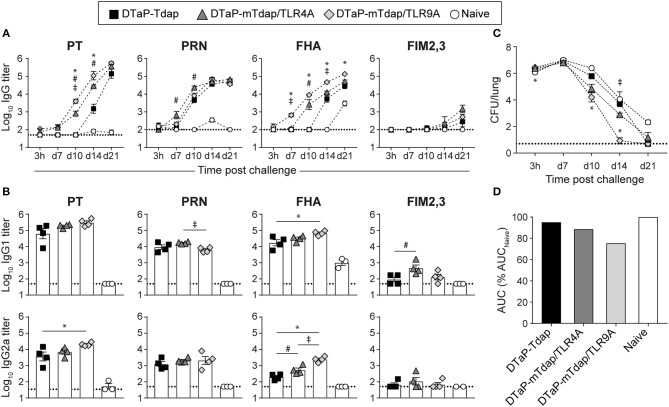
Boosting DTaP memory responses with mTdap/TLR9A but not mTdap/TLR4A accelerates bacterial clearance. Naïve BALB/c mice were adoptively transferred with 50 × 10^6^ splenocytes of DTaP-primed mice prior to boosting with Tdap, mTdap/TLR4A or mTdap/TRL9A. Their splenocytes were transferred to naive recipients prior to intranasal challenge with Bp. **(A)** The kinetics of PT-, PRN-, FHA-, and FIM2,3-specific IgG antibody responses were assessed in sera collected at indicated time-points after challenge. The graphs show the mean Log_10_ IgG titers (± SEM) of individual sera (*n* = 3–4 mice per group). **(B)** Serum IgG1 and IgG2a titers were assessed 21 days after challenge. The graphs show the mean Log_10_ IgG1 and IgG2a titers (± SEM) of individual sera (*n* = 3–4 mice per group). The dotted lines indicate the 50% cut-off of the assay. **(C,D)** Lungs were harvested 3 h and at days 7, 10, 14, and 21 after challenge for determination of bacterial colonization. The graphs show **(C)** the Log_10_ number of CFUs per lung (± SEM) at the indicated time-points for *n* = 3–4 mice per group and **(D)** the area under the clearance curve (AUC) normalized the AUC from naïve mice. Data are representative of at least two independent experiments. *P*-value < 0.05 for: ^*^DTaP-Tdap vs. DTaP-mTdap/TLR9A; #DTaP-Tdap vs. DTaP-mTdap/TLR4A; ‡aP-mTdap/TLR9A vs. aP-mTdap/TLR4A.

For the experiments of Figure 1, *Bordetella pertussis* 18323 (provided by the US FDA) was grown on Bordet-Gengou agar (Difco) supplemented with 1% glycerol, 20% defibrinated sheep blood (Sanofi Pasteur, Alba La Romaine). 5 × 10^6^ colony-forming units (CFU) were instilled intranasally in a volume of 30 μl into mice anesthetized by intramuscular injection of Imalgen (ketamine 60 mg/kg; Merial SAS) and Rompun (Xylaxine 4 mg/kg; Bayer). For experiments shown in Figures 2–**5**, streptomycin-resistant *Bordetella pertussis* Tohama I derivative BPSM, a kind gift from Prof. Camille Locht (Institut Pasteur, Lille) (38), were grown on Bordet-Gengou agar (Difco) supplemented with 1% glycerol, 10% defibrinated sheep blood (Chemie Brunschwig AG) and 100 μg/ml streptomycin. 1 × 10^6^ colony-forming units (CFU) were instilled intranasally in a volume of 20 μL into mice anesthetized by intraperitoneal injection of Ketasol (100 mg/kg; Graeub) and Rompun (10 mg/kg; Bayer). Mice were sacrificed 2–3 h after infection for quantification of the initial numbers of viable Bp CFUs in the lungs and at different time-points post challenge for determination of bacterial colonization. Briefly, lungs homogenates were plated onto Bordet-Gengou agar plates and the number of CFUs was counted after 4 days of incubation at 37°C. Protective efficacy (reflected by bacterial clearance) was expressed as the area under the clearance curve (AUC) value normalized to that of naive control mice (ΔAUC).

### Antibodies Quantification

In the experiments shown in Figure 1, pertussis antigen-specific IgG1 and IgG2a antibodies were titrated in a multiplex MSD U-PLEX assay (Meso Scale Discovery). The coating proteins were coupled to biotin to allow their subsequent coupling to the linkers present in the bottom of Uplex plate. Uplex plates were coated with PT (2 μg/ml), PRN (10 μg/ml), FHA (3 μg/ml), FIM2,3 (4 μg/ml), DT (4 μg/ml), or TT (8 μg/ml) (all antigens from Sanofi Pasteur). Serial dilutions of serum sample, control and reference sera (WHO/NIBSC reference Bp anti-serum (NIBSC code: 97/642) for IgG1 and an in-house pool of hyperimmune sera for IgG2a) were added, a wash step performed, and IgG1 or IgG2a antibodies bound to each antigen were detected using anti-IgG1 or anti-IgG2a (Jackson ImmunoResearch) antibodies linked to SULFO-TAG™ (RD-Biotech) using MSD GOLD SULFOTAG NHS-Ester Conjugation kit (Meso Scale Discovery).

In the experiments shown in Figures 2–**5**, Bp antigen-specific antibody titers were determined by ELISAs. Briefly, 96-well plates (Nunc MaxiSorp™; ThermoFischer Scientific) were coated with PT (1 μg/ml), PRN (5 μg/ml), FHA (5 μg/ml), or FIM2,3 (2 μg/ml). Wells were incubated with 2-fold serial dilutions of individual or pooled mouse prior to incubation with secondary horseradish peroxidase (HRP) conjugated anti-mouse IgG, anti-mouse IgG2a (both from Invitrogen), and anti-IgG1 (BD PharMingen). The optical density of each well was measured at 405 nm and the data analyzed with SoftMax Pro software. IgG, IgG1, and IgG2a titers were expressed as Log10 in reference to the WHO/NIBSC reference Bp anti-serum (NIBSC code: 97/642) (IgG, IgG1) or in reference to a titrated pool of hyperimmune sera (IgG2a).

### Statistical Analysis

Values are expressed as mean ± SEM. Statistical analysis were performed using unpaired *t*-test or one-way ANOVA followed by a Tukey multiple comparison test when more than two groups of mice were tested. All analysis were done using the Prism 7.0 (GraphPad software). Differences with *p* > 0.05 were considered insignificant.

## Results

### Modified Acellular Pertussis Vaccines Protect Efficiently Against Pertussis Challenge Independently of TLR4A or TLR9A Supplementation

We first tested the capacity of three novel mTdap formulations to boost immune memory elicited by current DTaP vaccines. CD1 mice were primed i.m. with DTaP and boosted 42 days later with mTdap with/without TLR4A or TLR9A. A control group was primed and boosted with DTwP, known to better protect than DTaP (6, 7) (see Table 1 for abbreviations and vaccine content).

DTaP/mTdap elicited similar titers of PRN-, FHA-, and FIM2,3-specific IgG1 and IgG2a antibodies as DTwP/DTwP and higher PT-specific IgG1 6 weeks after boosting (Figure 1A), in line with the lower PT content of DTwP (39). The addition of TLR4A or TLR9A to mTdap did not significantly affect antibody titers (Figure 1A) nor their IgG1/IgG2a ratio (data not shown). T cell responses were also assessed 42 days after boosting for the secretion of Th2- (IL-5), Th1- (IFNγ), and Th17- (IL-17) cytokines. In line with the respective Th2- and Th1-inducing properties of aP and wP vaccines (16, 20), mTdap significantly induced IL-5-secreting splenocytes whereas DTwP preferentially induced IL-17- and IFNγ-producing cells, although differences in IFNγ did not reach statistical significance in this experimental setting (Figure 1B). Compared to mTdap, mTdap/TLR4A and mTdap/TLR9A formulations significantly reduced IL-5 responses (to similar levels as DTwP), without increasing IFNγ-producing cells. IL-17-producing cells were only observed after mTdap/TLR4A boosting, reaching similar numbers as in DTwP-primed/boosted mice (Figure 1B). DTaP/mTdap elicited similar antibody and T cell responses as DTaP/Tdap (data not shown).

To evaluate the protective efficacy of mTdap-based boosters through bacterial clearance, mice were challenged intranasally with Bp 42 days after boosting. Bacterial loads remained high in the lungs of naïve mice, initially increased and started to decrease after day 3 (Figures 1C,D). In contrast, rapid bacterial decline was observed in the lungs of all immunized mice. By day 3, bacterial colonization was slightly but significantly lower in mice primed and boosted with mTdap formulations (with/without TLR4A or TLR9A) compared to DTwP, as previously reported (40). Nevertheless, by day 7, most of the mice had cleared the infection (Figures 1C,D). DTaP/mTdap elicited similar protection as DTaP/Tdap (data not shown).

Thus, the conventional murine model only discriminated Tdap, mTdap or TLR4A/TLR9A-containing mTdap formulations by the down-regulation of IL5/Th2 responses (mTdap/TLR4A or TRL9A) and the induction of Th17 responses (mTdap/TLR4A).

### An Adoptive Transfer Model of Pertussis Immunity to Better Recapitulate the Human Situation

The rapid protection conferred in mice by DTaP/DTwP priming and mTdap/DTwP boosting may reflect the contribution of both pertussis-specific antibodies and T cell effectors present at time of challenge (Figure 1). In humans, however, vaccine-induced antibodies rapidly wane and are frequently low or absent at time of exposure by boosting or infection. To mimic this condition and assess in mice the protective efficacy of novel vaccine formulations in the absence of circulating antibodies, we developed adoptive transfer models. Following upon our single adoptive transfer model, which enables the characterization of the influence of priming (37), we developed here a double transfer model to assess the influence of boosting—both in absence of serum antibodies (Figure 2A).

Despite the use of distinct mouse strains, bacterial strains, and experimental procedures (anesthesia, etc.) in Lyon/France and Geneva/Switzerland, similar antibody responses and bacterial clearance patterns were observed both in naïve and immunized mice [Figures 1C,D, 2E and (37)]. This allowed using and further developing in BALB/c mice the adoptive transfer model developed in Geneva.

To establish the benchmarks with current vaccines, we primed mice with DTaP or DTwP and transferred 50 × 10^6^ splenocytes into naïve recipient BALB/c mice—subsequently boosted with Tdap or DTwP. After priming, anti-PT, FHA, PRN, and FIM2,3 IgG antibodies were similar as observed in CD1 mice (Figure 2B; Supplementary Figure 1). Six days after the first adoptive transfer (d-1), PT-, PRN-, FHA-, and FIM2,3-specific serum antibodies were undetectable in recipient mice, as wished (Figure 2C). Tdap and DTwP boosting rapidly reactivated antibody responses in recipient mice, with detectable PT-, PRN-, FHA-, and FIM2,3-specific IgG antibodies from 7 days onwards and reaching a plateau by day 14 (Figure 2C). We previously reported that the Tdap boosting of non-transferred naïve mice induces much lower and slower kinetics of anti-PT antibody responses, which only appear by day 14 (37). While all prime/boost strategies similarly recalled FHA- and FIM2,3-specific responses, mice transferred with DTaP-primed cells developed faster and higher anti-PT IgG responses than recipients of DTwP-primed cells (Figure 2C), consistently with their primary responses to PT (Figure 2B). In contrast, mice transferred with DTwP-primed cells developed significantly more robust anti-PRN responses, independently of the boosting strategy (Figure 2C). This confirmed that the adoptive transfer of spleen memory cells preserve the relative ratio of antigen-specific primed cells.

To evaluate the protective efficacy of booster formulations in the absence of circulating antibodies at time of challenge, we performed a second adoptive transfer into naïve BALB/c mice, 7 days prior to bacterial challenge. The kinetics of Ag-specific antibody responses was very slow in naïve mice: PRN- and FHA-specific IgG appeared by day 14, PT by day 21, and FIM2,3 IgG antibodies remained undetectable at all time-points (Figure 2D). As expected, recipients of memory cells raised faster antigen-specific responses: Bp infection mostly reactivated strong anti-PRN responses, and robust but slower anti-PT and FHA responses. FIM2,3-specific antibodies were detectable early but at low titers only in recipients of DTwP/DTwP immune cells (Figure 2D). Of note, the Tohama I Bp strain used here and in the following experiments expresses only the serotype 2 of FIM (41), explaining the very low or undetectable Ab titers for FIM2,3 after the challenge.

Lungs were collected at various time-points and analyzed for their bacterial content. In contrast to the early (day 3) reduction of bacterial counts observed when circulating antibodies are present at time of challenge (Figures 1C,D and data not shown in BALB/c mice), bacterial loads increased between day 0 and day 7 in Tdap-boosted recipient mice and Tdap boosting had no impact on Bp clearance, independently of DTaP or DTwP priming. In contrast, a plateau of bacterial CFUs was observed on day 7 in DTwP/DTwP recipients, followed by a significantly faster clearance (Figures 2E,F): only all recipients of DTwP-boosted splenocytes had cleared bacteria by day 21 (data not shown).

In summary, this novel double-transfer adoptive model allows discriminating the ability of different boosting strategies to reactivate Bp immunity and to confer protection/enhanced bacterial clearance against challenge in the absence of confounding high titers of serum antibodies. In this model, DTwP/DTwP enhanced bacterial clearance but not TdaP boosting, thus validating the double-transfer model and benchmarking the optimal protective efficacy for novel booster candidates.

### A Single Dose of TLR4A- or TLR9A-Containing mTdap Enhances Pertussis Protection

mTdap, mTdap/TLR4A, and mTdap/TLR9A were similarly protective when used as boosters following DTaP priming in the conventional model (Figure 1). To investigate the immunogenicity and protective capacity of a single dose of mTdap/TLR4A and mTdap/TRL9A in the absence of serum antibodies, we immunized BALB/c mice with mTdap/TLR4A or mTdap/TLR9A 42 days before transferring their splenocytes into naïve mice. Both formulations rapidly induced PT-, PRN-, FHA-, and FIM2,3-specific IgG responses, starting on day 7 or 14 post immunization (Figure 3A). mTdap/TLR9A significantly enhanced IgG responses to PT and FHA compared to mTdap/TLR4A, despite slower kinetics (Figure 3A). mTdap/TLR4A induced predominantly IgG1 responses (Supplementary Figures 2A,B), resulting in significantly higher IgG1/IgG2a ratios compared to mTdap/TLR9A (Supplementary Figure 2C), consistently with the potential of TLR9 agonists to skew responses toward a Th1 profile (13, 28, 29).

Bp challenge recalled rapid and strong anti-PT, PRN and FHA IgG responses in recipients of immune cells (Figure 3B). Bacterial challenge better reactivated PT memory responses elicited by mTdap/TLR9A than mTdap/TRL4A, as shown by significantly faster and stronger IgG titers. Only minor differences were observed for PRN- and FHA-specific responses while anti-FIM2,3 IgG remained barely detectable (Figure 3B). Both adjuvanted formulations significantly enhanced bacterial clearance compared to naïve mice (Figure 3C). mTdap/TLR9A provided earlier bacterial control than mTdap/TLR4A, as shown by significantly lower bacterial load after day 10. However, the two formulations conferred similar protection at day 14 and all mice had cleared the infection by day 21 (Figure 3C), resulting in similarly smaller ΔAUC (mTdap/TLR9A: 64.7%; mTdap/TLR4A: 72.1%) compared to naïve mice (Figure 3D).

Thus, a single dose of either mTdap/TLR4A or mTdap/TLR9A induces potent memory responses that confer protection against Bp when reactivated in the absence of serum antibodies.

### Boosting DTaP With mTdap/TLR9A but not mTdap/TLR4A Favors a Th1-Associated IgG2a Antibody Profile Despite DTaP Priming

We next investigated whether these formulations remain sufficiently Th1-driving and thus protective in the Th2-skewed setting elicited by DTaP priming. To this end, we used the double adoptive transfer model described in Figure 2. Recipients of DTaP-primed splenocytes were boosted with Tdap (control), mTdap/TLR9A or mTdap/TLR4A. As the large number of mice required for these double adoptive transfer experiments did not allow the direct assessment of T cell responses, IgG1 and IgG2a titers were used as surrogates for Th2 and Th1-associated responses, respectively. Both adjuvanted mTdap formulations reactivated robust IgG responses reaching significantly higher titers than Tdap boosting (Figure 4A). Overall, antigen-specific IgG1 and IgG2a titers mirrored those of total IgG, and significantly higher IgG1 and IgG2a titers were observed following mTdap than Tdap boosting (Figure 4B). Interestingly, mTdap/TLR9A further increased IgG2a responses to PT, PRN, and FHA as compared to mTdap/TLR4A, resulting in a significantly smaller IgG1/IgG2a ratio for these three antigens (Figure 4C). Given the higher antigen content of mTdap vs. Tdap (Table 1), we first compared booster responses in our double adoptive transfer model. mTdap boosting elicited slightly but significantly higher PT and PRN titers than Tdap, likely reflecting the higher antigen content, but similar FHA and FIM2,3 antibody responses (Supplementary Figure 3A). Thus, when used in the absence of serum antibodies, mTdap-adjuvanted formulations designed to boost DTaP priming increase humoral responses, but only mTdap/TLR9A enhances Th1-associated IgG2a antibody responses in the context of DTaP-induced Th2 primary responses.

### Boosting DTaP With mTdap/TLR9A but not mTdap/TLR4A Enhances Protection Against Bp

As previously observed in Figure 2, Bp-induced responses remain extremely low/slow in naïve mice. In contrast, a faster and stronger reactivation of PT (day 10) and FHA (day 7) IgG responses were observed in recipients of DTaP-primed/mTdap/TLR9A-boosted cells as compared to recipient of DTaP/Tdap-boosted cells (day 14) (Figure 5A). Recipients of DTaP-primed/Tdap/TLR4A-boosted cells showed an intermediate phenotype with slower/lower PT and FHA responses (Figure 5A). Anti-PRN IgG responses were similar in all groups and anti-FIM2,3 IgG antibodies were again barely detectable (Figure 5A). In line with booster responses (Figure 4B), we observed a significant increase in PT- and PRN-specific IgG2a antibody titers in mice that received DTaP-primed/mTdap/TLR9A-boosted cells, despite overall low levels of IgG2a antibodies (Figure 5B). Again as previously observed (Figure 2E), the kinetics of bacterial clearance were similar between naïve mice and recipients of DTaP-primed/Tdap-boosted cells (Figure 5C). Despite slightly higher and faster PT and PRN antibody recalls, boosting with mTdap did not improve protection compared to Tdap (Supplementary Figures 3B–D), confirming that the currently used Tdap vaccine could be used as control. DTaP-priming/mTdap/TLR4A boosting conferred a slightly earlier bacterial control, reflected by significant lower bacterial counts on day 10, but with no overall impact compared to DTaP-priming/Tdap boosting (Figures 5C,D). In contrast, mTdap/TLR9A boosting after DTaP-priming significantly accelerated bacterial clearance (Figures 5C,D) to a similar extent than observed after a DTwP prime/boost schedule, as reflected by smaller ΔAUC (DTwP-DTwP: 83.5%; DTaP-mTdap/TLR9A: 75.25%) (Figures 2E, 5D).

In conclusion, TLR4A and TLR9A added to mTdap vaccines behave differently in the absence of circulating antibodies, a condition in which mTdap/TLR9A induces memory responses better recalled upon bacterial challenge and markedly enhancing bacterial clearance.

## Discussion

The shortcomings of current aP vaccines raise the need of third-generation pertussis vaccines. Given the importance of priming, efforts are currently dedicated to define how to best prime young infants against pertussis, inducing potent and long-lasting B and Th1/Th17 cell effectors and memory. However, licensing a novel infant vaccine will be most challenging given the resources required to demonstrate its safety, its efficacy, its non-interference on responses to other infant vaccines, and its sustained boostability. The development of new aP formulations proving better at boosting and/or redirecting aP-primed memory responses in adolescents and adults is thus an interesting approach. Using a model of adoptive transfer, we show here that despite DTaP priming, an alum-based Tdap booster vaccine including genetically instead of chemically-detoxified PT (in addition to FHA, PRN, and FIM2,3 antigens) and a TLR9 agonist enhances Th1-associated IgG2a responses, induces memory responses that are better recalled by Bp and enhances protection against Bp.

The correlates of Bp protection for pertussis vaccines are not well-defined. A critical role in mediating protection has been attributed to antibodies (42), also supported by the transfer of pertussis-specific maternal antibodies to newborns (43). However, several murine studies have demonstrated an important role for CD4^+^ Th1/Th17 cells in long-lasting protection (44, 45), and these are often considered as critical effectors for novel pertussis vaccines. Here we demonstrate the critical role of antibodies, which rapidly clear all bacteria if present at sufficient titers at time of challenge, in contrast with the much slower bacterial clearance (only initiated when antibodies appear) when serum antibodies are absent at time of challenge. Our adoptive transfer model thus strongly suggests that the sole reactivation of memory Th1/Th17 cells is not sufficient to protect mice against Bp, which also requires the reactivation of B cell memory into potent antibody-secreting cells.

Human studies have demonstrated the importance of the priming in imprinting lifelong vaccine-specific T cell responses, as illustrated by the persistence of wP-induced Th1/Th17 polarization despite repeated aP boosters (46, 47). However, Bp challenge can boost and shift aP-induced immune responses toward Th1 response (15). Considering the important cohort of aP-vaccinated subjects worldwide, the identification of formulations able to redirect aP-driven Th2 responses toward Th1/Th17 represents an important milestone for the development of novel booster vaccines. In this study, we assessed alum-based formulations complemented with TLR4A or TLR9A as (1) studies in TLR4-deficient mice have identified the contribution of TLR4 signaling to the immunogenicity of wP vaccines (48) and protective immunity against Bp infection (49) induced by aP or wP immunization (23, 40), and (2) TLR9 signaling is known to promote Th1 responses (50). Importantly, TLR agonists have already been included in human vaccines currently licensed [MPL/TRL4 (51) and CpG/TLR9 (52)].

Consistently with previous data (13, 14, 26), both TLR ligands reduced the number of IL-5-producing T cells. This did not correlate with increased number of IFNγ-producing cells, and only TLR4A-based formulations elicited IL-17-secreting cells. The induction of Th17, but not Th1 cells, by TLR4A and not TLR9A is consistent with previous studies using a meningococcal LPS as TLR4 ligand in combination with alum (14), or CpG as TLR9 ligand in substitution of Alum (13) in aP formulations. The role of TLR4 signaling in Th17 cell responses has been demonstrated in TLR4-deficient mice, which showed impaired IL-17 secretion upon wP but not aP immunization (23). Thus, Bp LPS, present in wP formulations, is a key factor in its induction of Th17 responses.

The failure of TLR9A to enhance Th1 immune responses, which contrasts with two previous reports (13, 29), may have several explanations. First, none of these two studies demonstrated the effective induction of Th1 responses by TLR9A-based formulations in an aP-primed Th2-biased setting—i.e., following aP priming. Second, Ross et al. used CpG without Alum, thus avoiding the Th2-promoting intrinsic properties of Alum (13). Last, the C57BL/6 mouse strain used in the latter study is a prototypical Th1-prone mouse strain, in contrary to the more Th2-oriented mouse strains used here.

Based on decreased Th2 and increased Th17 responses (Figure 1), TLR4 signaling seemed more promising than TLR9 signaling at improving protection against Bp. However, mTdap/TLR9A slightly enhanced protection compared to mTdap/TLR4A when Bp challenge was performed after a single adoptive transfer. We observed significantly decreased IgG1/IgG2a ratios after a single dose of mTdap/TLR9A as compared to mTdap/TLR4A, indirectly suggesting that TLR9 signaling elicits stronger Th1-polarized responses than TLR4. The recall of PT and FHA antibody responses by Bp challenge was much faster in recipients of mTdap/TLR9A-primed cells. As protection relies mostly on the reactivation of memory B cells rather than T cells in the absence of circulating antibodies, this more rapid antibody response likely contributes to the better protective efficacy of mTdap/TLR9A.

mTdap/TLR9A showed potent efficacy after a single dose, or when given as a booster after the transfer of aP-primed splenocytes: this was reflected by higher and faster B cell memory recall and improved bacterial clearance. Lower IgG1/IgG2a ratios after boosting indirectly suggests that adding TLR9A to alum is able to redirect aP-induced Th2-associated IgG1 primary responses toward a more Th1-associated IgG2a profile. However, the observed changes are modest and direct analysis of T cell responses would be needed to confirm the extent of the ability of TLR9 ligands to redirect alum-induced pertussis-specific Th2 toward Th1 responses. This has been previously demonstrated upon neonatal/adult immunization against hepatitis B (53, 54), but not yet in the context of pertussis immunization.

The chemical treatment used in most current aP vaccines to detoxify PT is known to destroy many of its important protective epitopes (34), reducing the induction of neutralizing antibodies (35). By comparing the immunogenicity of various aP vaccines including chemically- or genetically-detoxified PT in infants, Edwards et al., clearly showed enhanced immunogenicity of the genetically-detoxified PT (55). Consistently, mouse studies showed that the gdPT used here generally exhibits higher immunogenicity than PT, especially when assessing neutralizing Ab titers (56). However, replacing PT by gdPT did not increase the protective efficacy of Tdap here. This may have two explanations. First in standard prime/boost murine model, the rapid clearance mediated by high antibody titers to all vaccine antigens likely masks any difference in the neutralizing ability of anti-PT antibodies. Second, in our adoptive transfer model, increased anti-PT antibodies were observed after boosting, confirming the higher immunogenicity of gdPT; however, booster-induced antibody responses do not contribute to protection, as the challenge is performed following a second adoptive transfer—in absence of circulating antibodies. This likely explains why the higher immunogenicity of gdPT is not reflected by improved protection in these murine models.

Although mice share multiple feature of pertussis disease with humans, they do not cough, they fail to transmit the disease to other mice, and they raise different lung pathophysiological responses (57). Using a murine model of intranasal infection, we show enhanced protective efficacy of the mTdap/TLR9A formulation, reflected by faster bacterial clearance. However, murine models may not be used to assess colonization and transmission, in contrast to non-human primates (NHP) (58) which develop similar symptoms of pertussis disease to humans (59). However, the NHP model does not permit to assess the respective contribution of memory T and B cell-mediated protection, as bacterial challenge is performed in presence of high levels of vaccine antibodies. Consequently, the conclusions raised from NHP studies may better apply to priming than to previously primed adolescent/adult vaccines who lost circulating antibodies. Our double adoptive transfer murine model overcomes this drawback, highlighting the importance of using diverse animal models to evaluate the various aspects of the protective efficacy of novel vaccines (60). Nevertheless, caution should be exerted when extrapolating from one species to humans, especially for adjuvanted formulations given differences in expression of TLR4/TLR9 (61, 62).

In conclusion, a double adoptive transfer murine model allows us to dissect the ability of different boosting strategies to recall Bp immunity and enhance bacterial clearance in the absence of circulating antibodies—a setting that resembles the human situation. It shows that the presence and/or rapid recall of pertussis antibodies are crucial to protection and that TLR9 (better than TLR4 agonists) may improve current aP vaccines and thus possibly better protect adolescents and adults against pertussis.

## Data Availability

The raw data supporting the conclusions of this manuscript will be made available by the authors, without undue reservation, to any qualified researcher.

## Ethics Statement

This study was carried out in accordance with the recommendations of Swiss and European guidelines and approved by the Geneva Veterinary Office and by French Ministry of Higher Education, of Research and Innovation and ethic committee.

## Author Contributions

FA, BM-G, MC-R, NR, MG, NM, YL, P-HL, MO, and C-AS designed the study. FA, BM-G, PF, MC-R, and NR performed the experiments. MG and NM manufactured and characterized vaccine formulations. FA, MB, BM-G, MC-R, NR, YL, P-HL, MO, and C-AS analyzed and/or interpreted the results. FA and C-AS wrote the manuscript. All authors contributed to manuscript revision, read and approved the submitted version.

### Conflict of Interest Statement

MC-R, NR, MG, NM, YL, and MO are employees of Sanofi Pasteur. The remaining authors declare that the research was conducted in the absence of any commercial or financial relationships that could be construed as a potential conflict of interest.

## Abbreviations

gdPT: genetically-detoxified pertussis toxin
PT: pertussis toxin
FHA: filamentous hemagglutinin
PRN: pertactin
FIM2, 3: fimbriae type 2 and 3
aP: acellular pertussis
wP: whole cell pertussis
Bp: *Bordetella pertussis*
TLR: Toll-like Receptor
TLR(4–9)A: Toll-like Receptor (4–9) agonist
Th: T helper
DC: dendritic cell.
