# Supplementary material for: Reactivating Immunity Primed by Acellular Pertussis Vaccines in the Absence of Circulating Antibodies: Enhanced Bacterial Control by TLR9 Rather Than TLR4 Agonist-Including Formulation
Source: Front Immunol. 2019 Jul 3;10:1520. doi: 10.3389/fimmu.2019.01520 (PMC6618515; doi:10.3389/fimmu.2019.01520)
Supplement: Supplementary file 1 [file Data_Sheet_1.pdf]

Supplementary Material

Supplementary Figures

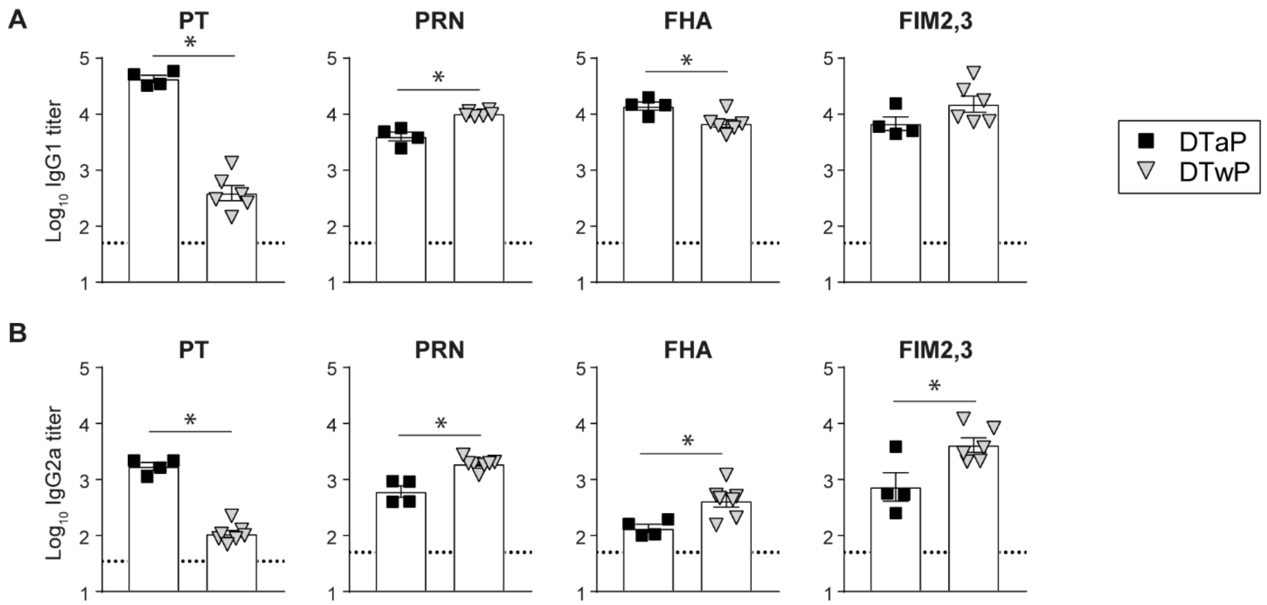

Supplementary Figure 1. Primary response to DTaP or DTwP vaccination. BALB/c mice were immunized i.m. with either DTaP or DTwP. Anti-PT, PRN, FHA, and FIM2,3 **(A)** IgG1 and **(B)** IgG2a antibody titers were assessed in sera collected 42 days later. The graphs represent the mean Log_{10} titers (± SEM) of pooled sera (n=4-7, 5-6 mice per pool). The dotted lines indicate the 50% cut-off of the assay. * P< 0.05.

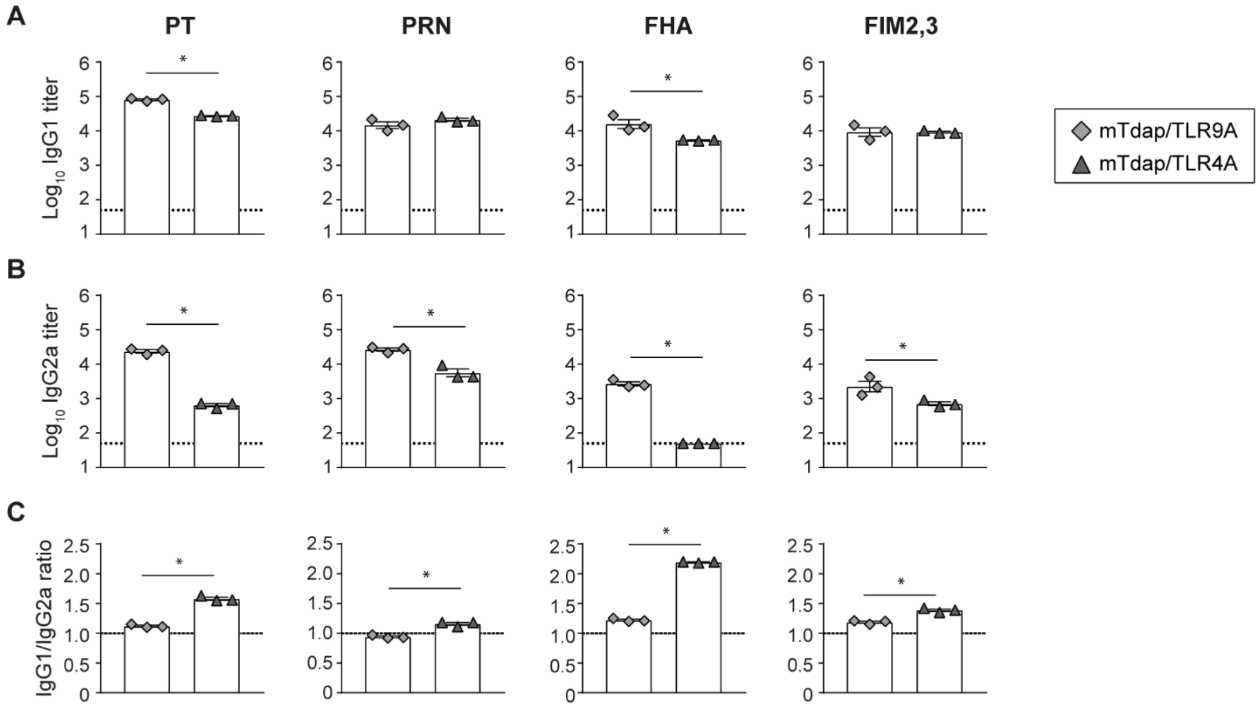

Supplementary Figure 2. Primary antibody responses to mTdap/TLR9A or mTdap/TLR4A immunization. BALB/c mice were immunized i.m. with mTdap/TLR4A or mTdap/TLR9A. PT-, PRN-, FHA-, and FIM2,3-specific IgG1 and IgG2a titers were assessed in sera collected 42 days later. The graphs show the mean Log10 (± SEM) of **(A)** IgG1 and **(B)** IgG2a titers and **(C)** the IgG1:IgG2a ratio of pooled sera (n=4, 6-7 mice per pool). The dotted lines indicate the 50% cut-off of the assay. * P < 0.05.

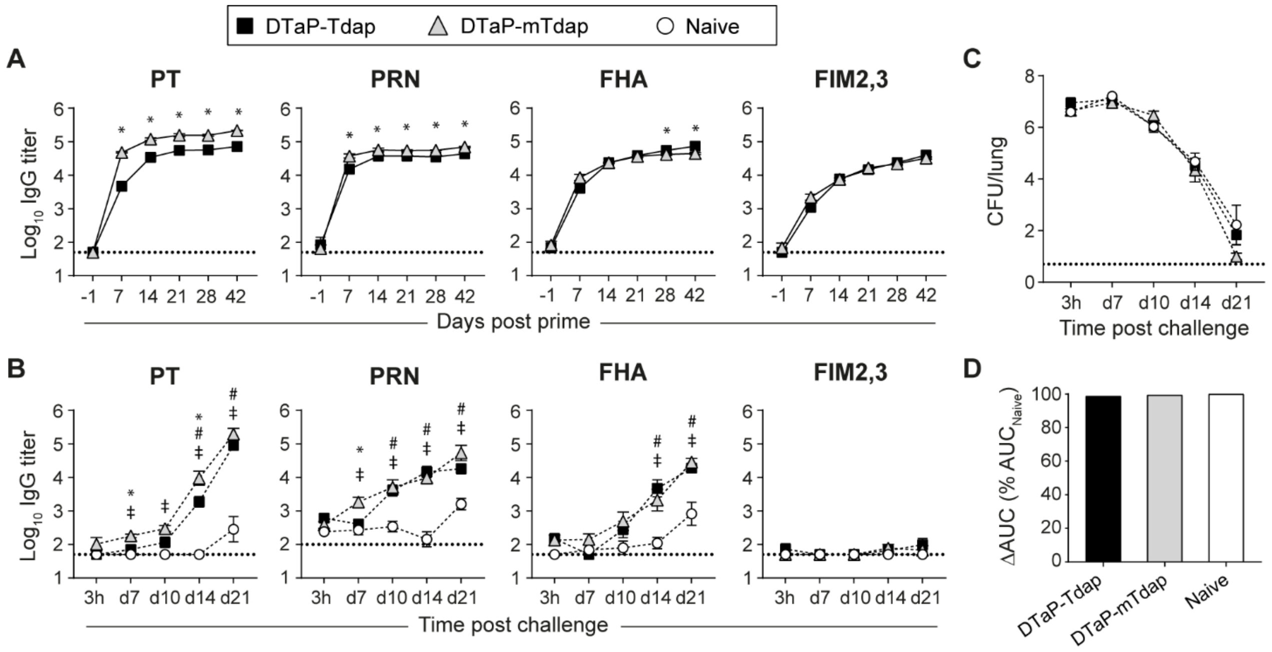

Supplementary Figure 3. Boosting DTaP memory responses with mTdap does not improve bacterial clearance. BALB/c mice adoptively transferred with 50x10^6 splenocytes of DTaP-primed mice received either Tdap or mTdap boosters. Six weeks later, their splenocytes were adoptively transferred into naïve BALB/c mice prior to intranasal challenge with Bp. PT-, PRN-, FHA-, and FIM2,3-specific IgG antibody responses were assessed in sera collected at indicated time-points after **(A)** the boost and **(B)** the challenge. The graphs show the mean Log_{10} IgG titers (± SEM) of **(A)** pooled (n=4, 6-7 mice per pool) or **(B)** individual sera (n=3-4). The dotted lines indicate the 50% cut-off of the assay. **(C-D)** Lungs were harvested at the indicated time-points after challenge for determination of bacterial colonization. The graphs indicate **(C)** the Log_{10} number of CFUs per lung (± SEM) at indicated time-points for n=3-4 mice per group and **(D)** the area under the clearance curve (AUC) normalized to the AUC from naïve mice. $P < 0.05$ for * DTaP-Tdap versus DTaP-mTdap; # DTaP-Tdap versus naive; ‡ DTaP-mTdap versus naive.
